# Water Potential from Adaptive Force Matching for Ice and Liquid with Revised Dispersion Predicts Supercooled Liquid Anomalies in Good Agreement with Two Independent Experimental Fits

**DOI:** 10.1021/acs.jpcb.3c06495

**Published:** 2024-03-27

**Authors:** Raymond Weldon, Feng Wang

**Affiliations:** Department of Chemistry and Biochemistry, University of Arkansas, Fayetteville, Arkansas 72701, United States

## Abstract

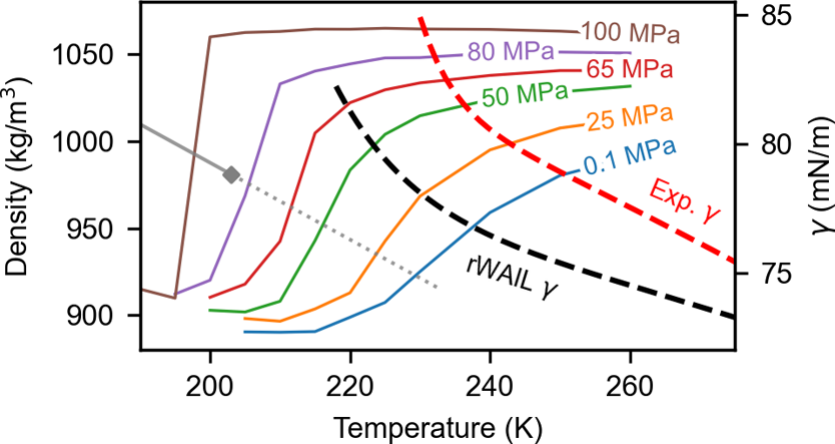

A revised version of the Water potential from Adaptive force matching for Ice and Liquid (WAIL) was developed by using the previous data set for fitting the WAIL model but with a dispersion term calculated using symmetry adapted perturbation theory (SAPT). The model has no adjustable parameters and relies solely on fitting first-principles information. The new model, named revised WAIL (rWAIL), shows improved predictions of most properties of water when compared to the previously published WAIL model. The rWAIL model also compares favorably to other first-principles-derived water models, such as MB-Pol, at only a fraction of the computational cost. The rWAIL model is used to study the properties of supercooled water. The model shows evidence of a liquid–liquid phase transition (LLPT) in the supercooled regimes with the liquid–liquid critical point (LLCP) at 203 K and 90 MPa. This estimate is in good agreement with a recent polynomial fit to the experimental density of water. Also, the fit to the surface tension of supercooled water based on the rWAIL model shows excellent agreement with the corresponding fit to the experimental data. Consistent with previously published molecular dynamics and experimental data, the surface tension of water exhibits exponential growth in the supercooled regime, which is likely a result of the emergence of a low-density liquid form of water. The simulation thus unites two separate experimental fits with one first-principles-based model, lending strong evidence of an LLPT in real water.

## 1 Introduction

Life on Earth is indebted to the existence of the temperature of the maximum density of liquid water above the melting temperature of ice. A promising theory that explains such a density anomaly predicts the existence of a liquid–liquid phase transition (LLPT) in the supercooled region.^1−4^ While several experiments showed evidence of two amorphous metastable forms of deeply cooled glassy water,^5−7^ most of the work in the so-called no man’s land, where ice nucleation is unavoidable even on relatively short time scales, is in the realm of molecular dynamics. Poole et al. were the first to suggest the existence of LLPT with simulations using the ST2 water model.^8^ Since their seminal work, several water models have been shown to exhibit a two-phase behavior in the supercooled regime with different estimates of the location of the liquid–liquid critical point (LLCP).^9−11^ For example, the ST2 model has an estimated LLCP at 245 K and 180 MPa,^12^ whereas TIP4P/Ice has an LLCP at 188 K and 174 MPa.^9^

A complication in LLCP predictions arises as various water models that do show LLPT behavior have different melting temperatures (*T*_M_). For example, ST2 melts at 300 K,^13^ whereas TIP4P/Ice has a *T*_M_ of 273 K by design. Thus, not only do the LLCPs for the different water models occur at different locations but they also have different degrees of supercooling. The high-density and low-density liquids are believed to have similar local structures as normal liquid and ice,^14^ respectively. Thus, the location of the LLCP will depend on the relative stability of the ice and liquid described by each water model.

Most empirical water models were fitted to the properties of the liquid only; such models tend to underestimate the *T*_M_ of ice.^15^ To the best of our knowledge, only two empirical water models have *T*_M_ values within 20 K of the experimental value: The TIP4P/Ice model,^16^ which was fitted to give the correct *T*_M_, and the TIP5P model,^17^ which was fitted to reproduce the temperature of maximum density. However, the TIP4P/Ice model shows too much structure for the liquid water, and the TIP5P model has a competing solid phase that is more stable than ice-Ih.^18^ The normal ice is thus metastable with TIP5P water.

One advantage of empirical water models is their simplicity, which allows microsecond scale trajectories to be simulated on typical workstations. More sophisticated ab initio-based water models also exist. For example, the successful MB-Pol model^19^ shows good accuracy by explicitly modeling polarization and other many-body effects; the model provides good agreement with experimental properties over a broad range of temperatures and pressures.^20,21^ At the same time, such models require quite substantial computational costs and frequently require specialized software for simulation.

The Water potential from Adaptive force matching for Ice and Liquid (WAIL) model^22^ is an ab initio derived model that uses energy expressions common to empirical force fields. The computational cost of rigid variants of the WAIL model is similar to that of TIP4P. The WAIL model was produced with adaptive force matching (AFM)^23^ by fitting to coupled cluster quality forces obtained using the density functional theory-supplementary potential (DFT-SP) approach.^24,25^ The WAIL potential was not empirically adjusted to reproduce the properties of any phase of water. At the same time, WAIL gives accurate descriptions of the properties of both ice and liquid with a *T*_M_ of 270 K.

The WAIL model has been shown to exhibit a two-phase behavior in the supercooled regime^26,27^ with an LLPT at 50 MPa and 207 K based on the estimate of Li and Wang.^27^ Moreover, the work by Rogers and Wang showed a clear correlation between the LLPT and the emergence of an exponential component in the surface tension of supercooled water.^28^ Although the physical origin of the exponential component has not been fully understood, the behavior was later confirmed by experimental measurements of the surface tension of real water by Vinŝ et al.^29^

Although the WAIL model may provide quite reliable estimates of the properties of supercooled water, the WAIL model has a limitation in that it cannot be used with long-range van der Waals corrections to pressure. More recent AFM models fit dispersion separately to symmetry adapted perturbation theory (SAPT)^30^ or Grimme’s empirical formalism for DFT.^31^ The WAIL model was developed by fitting the dispersion along with other parameters in the quantum mechanics/molecular mechanics (QM/MM) step of AFM. AFM generally uses small QM clusters. Getting a reliable description of the asymptotic behavior of a dispersion at long ranges is difficult when fitting only to small clusters.

In this work, we refit the WAIL model using the original set of conformations and reference forces except for using SAPT-based dispersion. It is shown that the revised WAIL model, rWAIL, predicted most properties in better agreement with experiments than the original WAIL model. The improved model gives the location of the liquid–gas critical point in better agreement with experiments. At low temperatures, the predictions of the rWAIL model are consistent with two separate fits to experimental specific density and surface tension regarding supercooled water.

The study will be presented in 5 sections. The fitting of the rWAIL potential is discussed in Section 2. The computational details of the properties are presented in Section 3, followed by results and discussion in Section 4 and the summary and conclusions in Section 5.

## 2 Fitting of the rWAIL Model

The AFM procedure^32^ fits molecular mechanics force fields using only reference QM information without any adjustable parameters. The models are fitted with the sole objective of reproducing the reference electronic structure potential energy surface. In this sense, simulations using AFM models can be understood as a more efficient method for performing ab initio MD.

Recent studies with AFM show that many experimental properties, such as hydration free energies, can be predicted reliably by AFM models based solely on electronic structure theory.^33−35^ Several water models have been developed using AFM differing in reference methods and training sets during AFM iterations.^36^ The B3LYPD-4F^37^ model fits to reference quantum mechanics (QM)/molecular mechanics (MM) forces computed with dispersion-corrected B3LYP. This model gives a high *T*_M_ of 287 K for ice, which is consistent with prior ab initio MD simulations showing that DFT tends to overestimate the *T*_M_ of water.^38,39^ Both BLYPSP-4F and the WAIL potential were fitted to a coupled cluster quality reference surface, computed using the DFT-supplemental potential (SP) approach.^24,25^ While the BLYPSP-4F model only included liquid water in the training set, the WAIL model has both ice and liquid as training set conformations. This results in a substantial improvement of the *T*_M_ for the WAIL model when compared to BLYPSP-4F.

Of the various water models developed in the early stages of AFM, the WAIL potential was created without a separate step to determine dispersion. Subsequent AFM studies suggest that using a separate dispersion fitting step, where the dispersion parameters are fitted to either SAPT or Grimme’s empirical formula with gas phase dimers^37^ or oligomers,^33^ will lead to a more reliable model. Although such an approach ignores many-body effects in dispersion, it has the advantage of improved numerical stability. When dispersion parameters are fitted together with all other parameters during AFM iterations, the dispersion term could be borrowed to fortuitously reduce inaccuracies in other types of stronger interactions at short ranges, such as Coulombic and exchange repulsion. Also, although the QM clusters for fitting the WAIL model contain 17 QM water molecules, a majority of these water molecules reside at the QM/MM boundary and are not used for fitting. Only water at the core of the QM region is fitted in AFM. The very small core also causes problems in determining the long-range tail of dispersion, which strongly affects the density of water.^40^

Although the WAIL model has shown adequate performance, the agreement deteriorates when long-range corrections to dispersion are applied. We thus refit the WAIL model using SAPT-based dispersion, as done in more recent AFM models. The exact fitting procedure of the revised model follows the same steps of the original WAIL model, which has been discussed in detail by Pinnick et al.^22^ with the only difference being a SAPT-based dispersion coefficient of −610.578 Å^6^ kcal/mol in the new model.^37^ This number was obtained by fitting it to the SAPT E^20^ dispersion of water and has been used in several AFM water models in the past.

The new model will be termed revised-WAIL (rWAIL), which uses energy expressions identical to the WAIL model. Although the energy expressions have been reported in the past,^22^ they are briefly summarized below for convenience.

The intermolecular interaction between water molecules is modeled by
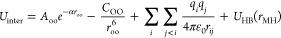
1where *r*_OO_ is the oxygen–oxygen distance and *r*_*ij*_ is the distance between charge sites *i* and *j* on two molecules. The function *U*_HB_(*r*_MH_) may be written as
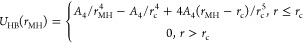
2where *r*_MH_ is the intermolecular MH distance.

The M site is the site that carries the negative charge and is defined using the two O–H bond vectors with the equation
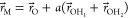
3where *r⃗*_O_ is the vector defining the O location, and the *a* parameter is shown in Table 1.

**Table 1:** Parameters for the Flexible and Rigid Versions of the rWAIL Model

parameter	rWAIL		
	rWAIL^a^	EG298^b^	EG273^b^
*q*_M_ (e)	–1.3464	–1.3464	–1.3464
*q*_H_ (e)	0.6732	0.6732	0.6732
*a*	0.2	0.2	0.2
*A*_OO_ (10^3^ kcal/mol)	240.9	240.9	240.9
α (Å^–1^)	4.098	4.098	4.098
*C*_OO_ (kcal Å^6^/mol)	610.578	610.578	610.578
*A*_4_ (kcal Å^4^/mol)	78.759	78.759	78.759
*r*_c_ (Å)	2.483	2.483	2.483
*r*_e_ (Å)	0.9507	0.9706	0.9709
*k*_2_ (kcal/(mol Å^2^))	1263		
*k*_3_ (kcal/(mol Å^3^))	–4831		
*k*_4_ (kcal/(mol Å^4^))	10,777		
θ_e_ (°)	106.66	105.25	105.34
*k*_θ_ (kcal/(mol rad^2^))	80.81		

a Flexible version of the model.

b Rigid version of the model created using the ensemble geometry at 273 K (EG273) and 298 K (EG298).

The intramolecular potential is modeled using quartic bonds and harmonic angles,
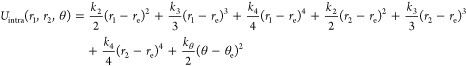
4where *r*_1_ and *r*_2_ are the two intramolecular OH distances and θ is the HOH angle, and the parameters *r*_e_ and θ_e_ are equilibrium bonds and angles. Note that for rigid models there is no intramolecular term and the *r*_e_ and θ_e_ are the bonds and angles being restrained.

All the parameters for the rWAIL model are provided in Table 1. We also list the parameters for two rigid variations of the rWAIL model that are optimized for simulations near 273 and 298 K, respectively. The difference between the two rigid models are only in their geometry as uniquely defined by *r*_e_ and θ_e_.

By eliminating the high frequency O–H vibrations, the rigid models allow 2 fs time steps to be used. This allows for much reduced computational costs, especially for simulating supercooled water. It has been shown previously that the rigid version of a flexible model should be created using ensemble-averaged geometry rather than the geometry of the model.^41^ Thus, the rigid models are also termed ensemble geometry (EG) models. The ensemble geometry depends on the temperature. The EG273 model, which uses the 273 K geometry, is more appropriate for simulating systems at lower temperatures close to *T*_M_ and the EG298 model, which uses the 298 K geometry is more appropriate for room temperature properties. It is worth noting that prior studies showed properties simulated with the EG models show better agreements with experiments when compared to the flexible counterpart.^41^ This has been attributed to an implicit consideration of quantum nuclear effects.

Gromacs input files for the flexible rWAIL and the EG273 and EG298 variants of the rWAIL model are provided in the Supporting Information for this study.

## 3 Computational Details

For creating the EG models, the geometries were averaged over 5 ns by using a box with 1728 flexible rWAIL water molecules at the corresponding temperatures and 1 bar. The geometry is defined by measuring the HH and OH intramolecular distances with MDTraj 1.9.7.^42^ Most properties of the normal liquid were simulated with the rigid EG298 version of the rWAIL potential. The *T*_M_ and the properties related to the supercooled water were computed with the EG273 model. Selected properties, such as diffusion constants, were computed using the flexible rWAIL model. The diffusion constant is one of the few properties where the EG model shows a fairly large difference from the flexible model, as shown in our previous study. We do not anticipate any major difference between the results of EG273 and EG298. This is confirmed by the heat capacity and surface tension computations at 298 K.

All simulations with the rigid models were performed with a 2 fs time step, except for *T*_M_. The *T*_M_ values were determined with the NVE three-phase coexistence method.^41^ A 0.5 fs time step was used for all the NVE simulations to ensure proper energy conservation. Flexible models were also simulated with a 0.5 fs time step.

All the simulations were performed with the Gromacs package version 2019.6.^43^ The SETTLES^44^ algorithm was used to maintain geometries for all rigid models. Unless mentioned otherwise, the following simulation parameters are used. The van der Waals interactions are cutoff at 1.0 nm, long-range corrections to energy and pressure are applied, and long-range Coulombic interactions are described with particle mesh Ewald.^45^ The system temperature is controlled with the Nose-Hoover thermostat^46,47^ with a relaxation time of 2 ps for all NPT simulations. The pressure in NPT simulations is controlled with the Parrinello-Rahman barostat^48,49^ with a 5 ps relaxation constant. NVT simulations are performed with the Nose-Hoover thermostat with a relaxation constant of 5 ps.

Properties calculated include the melting temperature (*T*_M_), radial distribution functions (RDFs), density as a function of temperature, surface tension (γ), dielectric constant (ε), heat capacity (*C*_p_), heat of vaporization (Δ*H*_vap_), diffusion constant (*D*), temperature (*T*_c_), pressure (*P*_c_), and density (ρ_c_) of the liquid–gas critical point, and the temperature (*T*_C_^LL^), pressure (*P*_C_^LL^), and density (ρ_C_^LL^) of the putative liquid–liquid critical point. The emergence temperature, *T*_e_, which is defined at the temperature where γ deviates from the standard IAPWS equation by the γ_s_ of 1 mN/m, is also computed by fitting to the IAPWS-E equation^28^
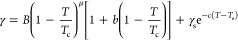
5

Just to clarify that γ was computed from a slab simulation using the standard formula,
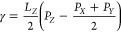
6where *L*_*Z*_ is the length of the box in the *Z* dimension, and *P*_*X*_, *P*_*Y*_*,* and *P*_*Z*_, are diagonal elements of the pressure tensor. The γ in a range of temperatures are computed with eq 6 and fitted to obtain *B, b,* and *T*_e_ according to eq 5. The *T*_c_ of this fit was taken from higher temperature liquid–vapor coexistence simulations using Wegner expansion. (*vide infra*)

All properties are listed in Tables 2 and 3 along with the models used to calculate such properties. The experimental values and properties of the WAIL models are also provided where available to allow easy comparison.

**Table 2:** Selected Properties of WAIL and rWAIL Water Models Compared to Those of Experiments and the MB-Pol Model

	*T*_M_ (K)	ε	γ (mN/m)	Δ*H*_vap_ (kJ/mol)	*C*_P_ (J/mol K)	*D* (10^–5^ cm^2^/s)	κ_T_ (10^–4^ MPa^–1^)
WAIL^41^	267 ± 1^a^, 272 ± 2^b^	85.6^b^	76.9^a^, 78.2^b^	53.4^a^	79.5^b^^,^^d^	1.53^a^	5.12^a^
rWAIL	265 ± 2^a^, 270 ± 1^c^	78.4^b^	70.9^a^, 71.2^b^, 71.6^c^	50.4^a^	80.7^b^, 80.7^c^	1.89^a^	5.74^a^
MB-Pol^57^	263.5	68.4	68.4	45.7	91.42	2.8	4.53
Exp.^58,59^	273.15	78.36	72.06	43.98	74.6	2.30	4.524

a The flexible version of each model.

b Simulated using the rigid version of the model created with the ensemble geometry at 298 K (EG298).

c Simulated using the rigid version of the model created with the ensemble geometry at 273 K (EG273).

d The heat capacity for WAIL as taken from ref (41) is *C*_v_ instead of *C*_p_.

**Table 3:** Liquid–Gas and Liquid–Liquid Critical Properties of the WAIL and rWAIL Models

	*T*_c_ (K)	*P*_c_ (bar)	ρ_c_ (kg/m^3^)	*T*_c_^LL^ (K)	*P*_c_^LL^ (MPa)	ρ_c_^LL^ (kg/m^3^)
WAIL^22,36^	711	125	286	207 (211)	50 (37)	(996)
rWAIL^a^	683	137	308	203	90	980
MB-Pol^60^	639	n/a	340	n/a	n/a	n/a
Exp.^58^	647.1	220.64	332.1	n/a	n/a	n/a

a Simulated using the EG273 version of rWAIL. The numbers in parentheses are from ref (26).

*T*_M_ was determined for both the flexible and the rigid EG273 version of the rWAIL model. The *T*_H_ and *T*_L_ used for the NVE three-phase coexistence method^41^ are 280 and 260 K, respectively, for the flexible rWAIL model, and 275 and 255 K, respectively, for the EG273 version.

Ambient surface tension was computed with the flexible EG298 and EG273 versions of the rWAIL model using a 1728 molecule slab in an orthorhombic box of dimension 3.58 × 3.58 × 10 nm^3^. The box thickness was chosen to ensure a 6 nm vacuum between the slab images. A Nosé–Hoover thermostat with a coupling constant of 2 ps was used. No long-range van der Waals correction was used for the slab simulation due to the inhomogeneity of the system. On the other hand, a long 1.75 nm van der Waals cutoff was used as recommended by prior studies to reduce errors associated with long-range van der Waals.^50^ The surface tension was measured over at least 15 ns of the trajectory.

γ at lower temperatures used to fit eq 5 were computed using the EG273 variant to better explore the supercooled regime. The simulation box contains 2139 molecules with dimensions of 3.999 × 3.999 × 10 nm^3^. Each slab was equilibrated for at least 10 ns before the γ was measured.

The ε was calculated with the EG298 version of the rWAIL model using the fluctuation dissipation theorem following the equation^51^
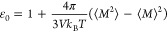
7

It should be noted that the static dielectric constant, ε, in Table 2 is scaled by the high-frequency dielectric constant of 1.78.^52^
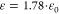
8

Variance of the dipole was measured over 5 ns at 298 K.

The Δ*H*_vap_ was calculated with the flexible rWAIL model using the following formula,
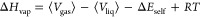
9where <*V>* is the average potential energy of the respective phase, and the self-energy term,^53^ Δ*E*_self_, is
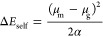
10

The ⟨*V*_gas_⟩ was found with a single molecule simulation under NVT conditions using a stochastic rescaling thermostat^54^ with a coupling constant of 0.5 fs at 298 K.

The heat capacity, *C*_p_, was calculated for the rigid models with the finite difference method using the equation
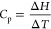
11where Δ*H* was measured using two NPT simulations at 295 and 301 K, for an average temperature of 298 K. The average enthalpy at each temperature was obtained from 15 ns of trajectory. The diffusion constant was found with the Einstein formula using 5 ns of NVT simulations at 298 K.

The liquid–gas critical properties were studied with the EG273 variant of rWAIL by simulating a slab of 2139 molecules at 8 different temperatures spanning the range from 325 to 625 K. Although the EG298 version or the flexible model would be more appropriate at such elevated temperatures, we used the EG273 model since one main focus of this study is the behavior of supercooled water. The IAPWS-E equation being fit uses the *T*_c_ as a parameter. We prefer to use *T*_c_ of EG273 to avoid any potential complications in the determination of *T*_e_.

The regions used to calculate the densities of the gas and liquid phase for the slab calculation were determined following the protocol of Hu and Wang.^50^ The liquid–gas *T*_c_ was determined by fitting the difference between liquid and vapor density with the Wegner expansion,^55^
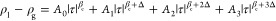
12where τ = 1 – *T*/ *T*_c_. β_c_ and Δ are chosen to be 0.325 and 0.5, respectively, according to the 3D Ising universality class. The critical density ρ_c_ was determined by fitting^56^
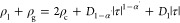
13

with the knowledge of *T*_c_ and an α^′^ value of 0.11 following previous work.^36,56^ The fit to the Wegner expansion is shown as Figure S1 in the Supporting Information.

The vapor pressure was measured as the normal pressure of the slab and fit using Antoine’s equation,
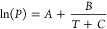
14

by minimizing the least-squares error of ln(*P*). After the *A, B,* and *C* parameters were determined from the fit, *P*_c_ was computed as the pressure at *T*_c_.

The putative liquid–liquid critical properties were determined by running NPT simulations using the EG273 version of the rWAIL model over a range of temperatures and pressures above 190 K and up to 1000 bar. Due to the need for long trajectories at low temperatures, all LLCP simulations were performed with a smaller cubic box containing 343 water molecules. The isothermal compressibility was calculated using the following equation
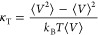
15

The κ_T_ under the ambient condition of 298 K and 1 bar is computed using the same formula with a 1728 water box and reported in Table 2.

## 4 Results and Discussion

**Figure 1:**
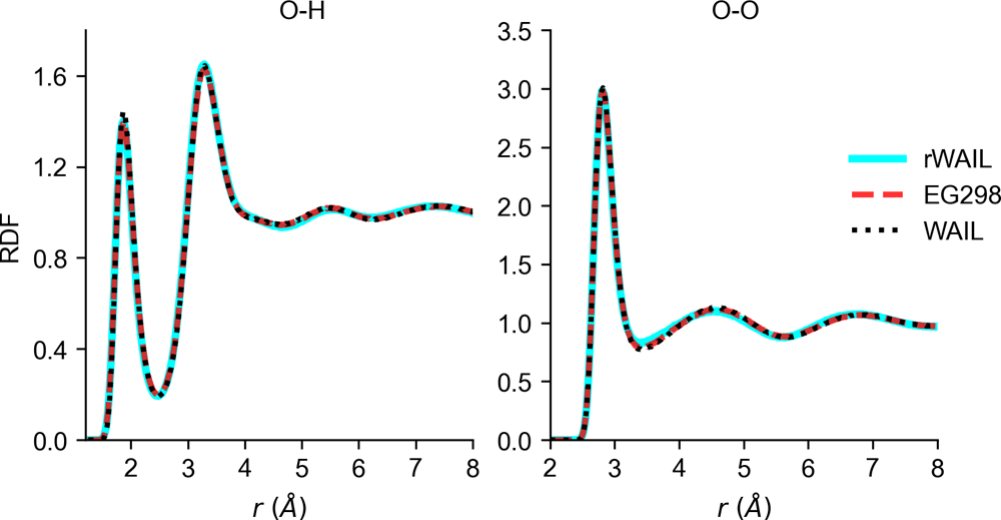
Radial distribution functions for the flexible versions of the WAIL, rWAIL models, and the EG298 variant of the rWAIL model.

Figure 1 shows the RDFs of the WAIL model, the rWAIL model, and the EG298 version of the rWAIL model at 298 K. As expected, with such a minor change of the dispersion parameter, even with all other parameters of rWAIL refitted, the RDF functions remain almost superimposable.

In Tables 2 and 3, we compare the properties predicted by WAIL and the new rWAIL model with experiments. We also list the values of the MB-Pol model taken from the literature.^57,60^ Although there are an abundance of water models to compare with, we pick MB-Pol because it is based on high-quality electronic structure calculations and is considered one of the most accurate water models.^61^

As seen in Table 2, the *T*_M_ of rWAIL decreased by approximately 2 K when compared to the *T*_M_ of the WAIL model computed using the same NVE three-phase coexistence method. For both models, the rigid variant produces a *T*_M_ approximately 5 K higher when compared to the flexible version, although the rigid version of the WAIL model uses the 298 K geometry, and the rigid variant of rWAIL uses the 273 K geometry. The difference between rigid and flexible versions of each model is larger than the difference between the WAIL and rWAIL models.

We argue that a rigid model could better represent real water due to nuclear quantum effects. With Newtonian mechanics, flexible water spends more time at the classical turning points of the O–H vibrations, whereas the O–H bond length in real water has a maximum probability at the value used in the rigid models according to quantum mechanics. The improvement is thus consistent with quantum effects being responsible for the rigid model giving better *T*_M_*.* In any case, the rWAIL model still gives excellent agreement with experimental *T*_M_, which was achieved without fitting to any experiments.

The 263.5 K *T*_M_ of MB-Pol computed using classical MD is slightly worse than the predictions of the WAIL and rWAIL models. We note that there are different ways to compute *T*_M_; the different methods could easily lead to a 2 K difference in *T*_M_. We thus believe the accuracy in predicting the ice Ih melting temperature of the MB-Pol model is comparable to that of the rWAIL. However, it is worth mentioning that the melting process can be simulated with rWAIL on a single workstation overnight, whereas comparable computations with MB-Pol currently will need a supercomputer with much larger scale parallelization.

The γ at 298 K is computed with all three versions of the rWAIL model. The rigid model shows a slight increase compared to the flexible model. The EG273 γ differs from EG298 γ by only 0.4 mN/m. Overall the rWAIL model provides a substantially improved γ when compared to the WAIL model. The WAIL model overestimates the γ value by 4–6 mN/m depending on whether molecular flexibility is modeled. The EG298 and flexible rWAIL underestimate γ by around 1 mN/m. It is worth noting that the truncation of long-range van der Waals for the slab calculations using the 1.75 nm cutoff is anticipated to lead to an underestimation of γ by around 1 mN/m under ambient conditions.^50^ Thus the minor disagreement of rWAIL could very well be a result of an insufficient van der Waals cutoff. The MB-Pol model underestimates γ by roughly 4 mN/m, which is in poorer agreement than that of rWAIL.

The ε of the rWAIL model also showed substantial improvement compared to the corresponding WAIL model, reaching almost perfect agreement with experiments. The ε of the MB-Pol model is roughly 10 below the experimental and rWAIL values, with the rWAIL model showing excellent agreement with experiments. The heat capacity of rWAIL showed slightly worse agreement when compared to WAIL although the difference between the two models is only 1.2 J/(K mol). It is worth noting that the heat capacity is a property substantially affected by nuclear quantum effects. While classical MD with a rigid body captures part of the quantum nuclear effects by removing intramolecular vibrations, it is impossible to judge how much of the difference from the experiment is a result of failing to account for the remaining nuclear quantum effects. Table 2 reports the *C*_P_ values of both the EG273 and EG298 models showing no measurable difference within the precision of our simulation. This is expected considering the EG298 and EG273 models only have very tiny geometry differences.

We note that the substantially higher *C*_P_ of MB-Pol does not indicate that the MB-Pol model provides a less accurate description of this property. The *C*_P_ of MB-Pol was computed as a flexible model with classical MD. The *C*_P_ of WAIL and rWAIL were computed using rigid variants of these models. The rigid variants suffer much less of the missing nuclear quantum effects since the high frequency vibration modes are eliminated.

Δ*H*_vap_ also shows a modest improvement of the rWAIL model compared to WAIL. Δ*H*_vap_ is another property that is strongly influenced by nuclear quantum effects. Path integral studies show that nuclear quantum effects are expected to reduce the magnitude of Δ*H*_vap_ by 6 kJ/mol.^62^ Thus, the rWAIL Δ*H*_vap_ computed with classical MD is expected to agree with experiments within 1 kJ/mol, assuming the 6 kJ/mol quantum correction is accurate. Before correction for quantum effects, the classical Δ*H*_vap_ of MB-Pol is in better agreement with experiments when compared to the rWAIL and WAIL models.

The *D* of the rWAIL model also showed substantial improvements when compared to WAIL. The original WAIL model gave a *D* of 1.53 × 10^–5^ cm^2^/s, with the improved model giving a value of 1.89 × 10^–5^ cm^2^/s. Path integral simulations have shown that the quantum corrections increase the *D* of water models with anharmonic bond terms by about 15%.^63^ A 15% correction will give a rWAIL *D* of 2.17 × 10^–5^ cm^2^/s, which is in good agreement with the 2.30 × 10^–5^ cm^2^/s experimental value. Even with a fairly large 1728 water box, we do anticipate the remaining finite size effects^64^ to cause the simulations to slightly underestimate the *D*, which is consistent with the observed difference. The *D* of 2.8 × 10^–5^ cm^2^/s of MB-Pol was extrapolated to infinite box size but without nuclear quantum correction. It seems to be too high if the 15% correction is to be applied.

The κ_T_ is a property that the rWAIL water showed substantially poorer agreement when compared to WAIL. As seen in Table 2, while the WAIL model overestimates the experimental κ_T_ by 13%, the rWAIL model overestimates experiments by 27%. For this property, the MB-Pol model is substantially better, showing perfect agreement at 298 K.^57^ The better agreement of the MB-Pol model may suggest that the κ_T_ is a property that is sensitive to explicit modeling of many-body effects; however, some empirical pairwise potentials also give good κ_T_. For example, the κ_T_ of TIP4*P*/2005 is 4.65 × 10^–4^ MPa^–1^.^65^ Although rWAIL shows some deficiency in quantitatively reproducing κ_T_, we do believe that the overall shape of the κ_T_ curve is not affected by the overestimation.

Properties related to the critical phenomena are listed in Table 3. The *T*_c_, *P*_c_, and ρ_c_ of the rWAIL model all show better agreement with experiments when compared to WAIL. We note that both the WAIL and rWAIL potentials were fitted to model condensed phases of water. No cluster conformations were present in the training set. In addition, the simple energy expressions would not be able to capture the change in many-body contributions from the condensed phase to the gas phase. Thus, it is expected that the models would underestimate the stability of the gas and give a critical point slightly too high and critical pressure too low. The MB-Pol model, by explicitly modeling many-body effects, indeed gives a better critical temperature and density, although it is also associated with substantially increased computational cost.

**Figure 2:**
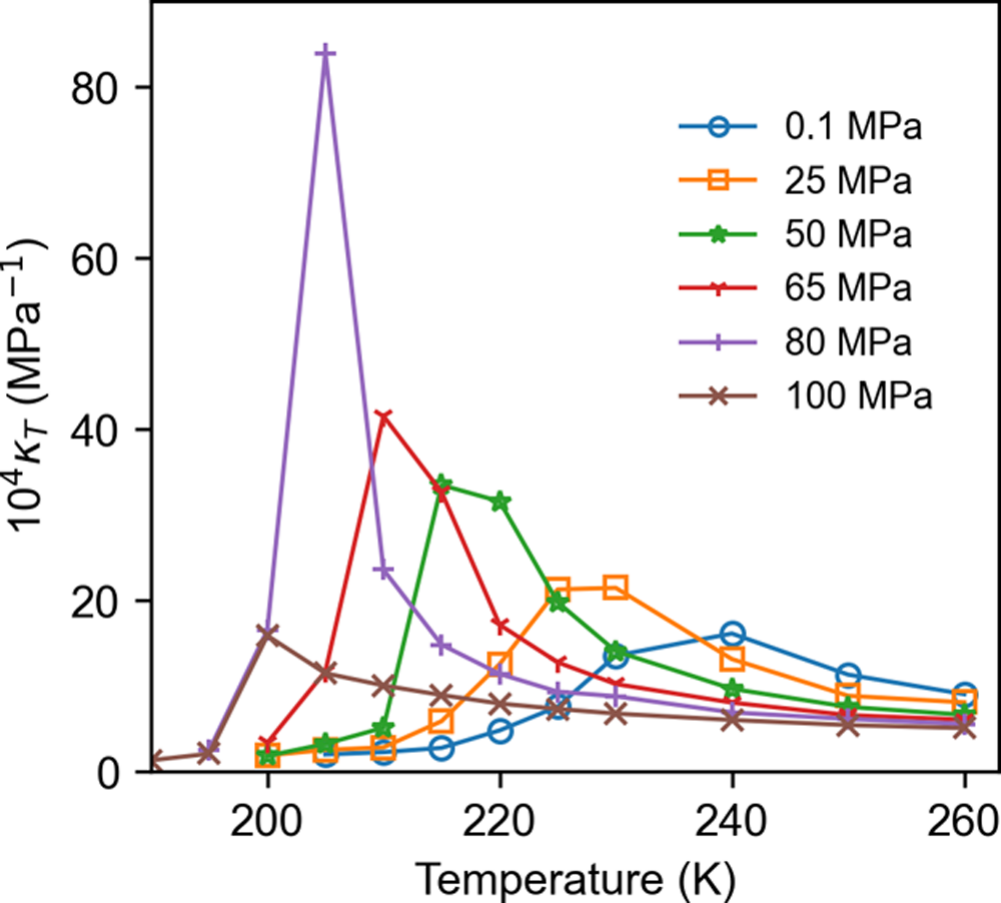
Isothermal compressibility as a function of temperature and pressure for the rWAIL model (computed with the EG273 variant).

**Figure 3:**
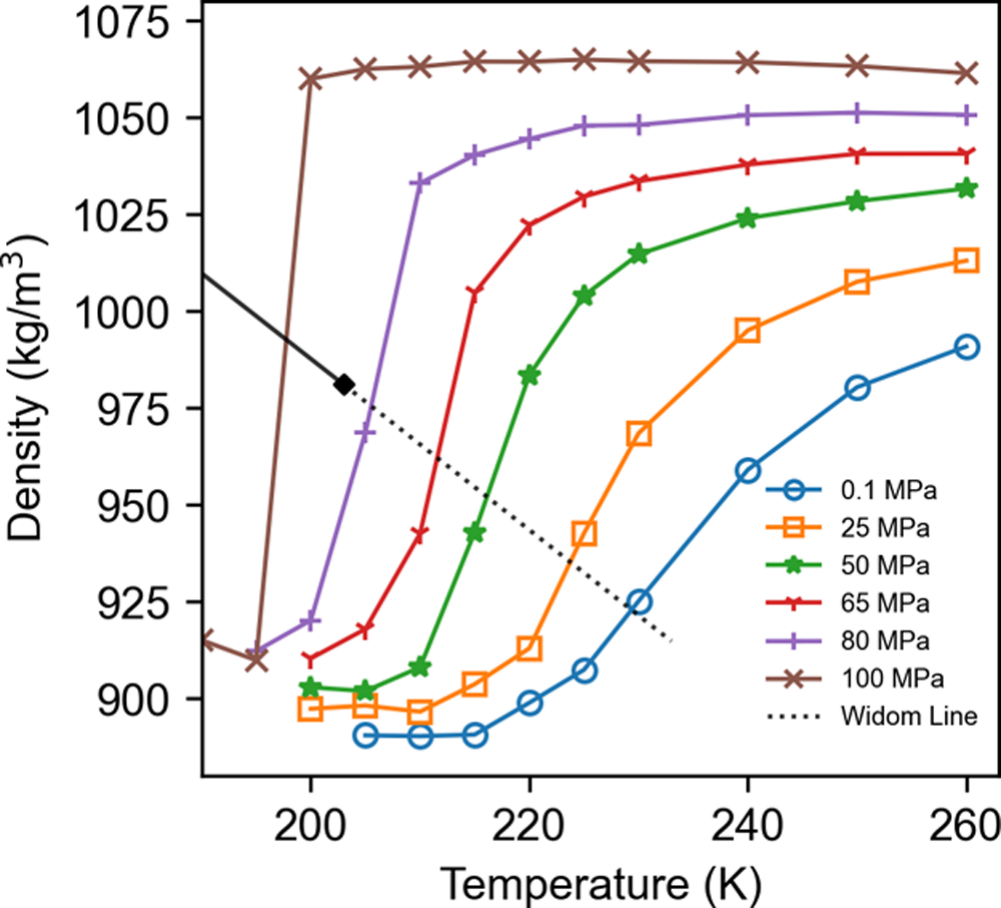
Density as a function of temperature and pressure for rWAIL (computed with the EG273 variant).

The putative LLPT in supercooled water is investigated by computing the κ_T_ (Figure 2) and density as a function of temperature (Figure 3). The critical point is associated with a divergence of κ_T_ and the density will show a jump across a first-order transition line but only a gradual change across the Widom line. It is clear the rWAIL water shows a sharp transition in density between 195 and 200 K at 100 MPa. While the κ_T_ shows a large peak around 205 K at 80 MPa, the peak significantly diminishes at 100 MPa, suggesting the putative LLCP to be above 80 MPa and close to 100 MPa. We thus estimate the LLCP critical point to be 203 K at 90 MPa with an uncertainty of ±5 K and ±10 MPa. While the 207 K *T*_C_^LL^ based on the WAIL model is within the uncertainty of the estimate with rWAIL, the rWAIL *P*_C_^LL^ is clearly higher than the 50 MPa value of the WAIL model.

**Figure 4:**
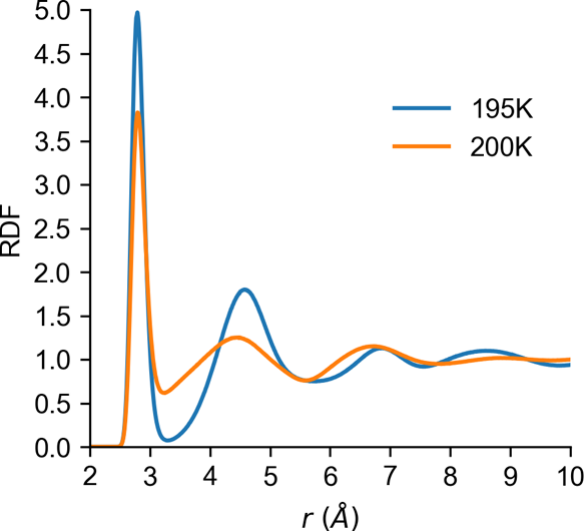
O–O radial distribution function for water at 195 and 200 K at 100 MPa according to the rWAIL model (computed with the EG273 variant).

Figure 4 shows a comparison of the RDF for water at 100 MPa across the LLPT line at 195 and 200 K. A sharp transition can be seen. Similar transitions have been observed previously with other water models.^66^ The low-density liquid (LDL) at 195 K has a much sharper first peak than that of the high-density liquid (HDL). More importantly, the second peak in the O–O RDF moves to a larger distance for the LDL. The LDL is clearly more structured and shows pronounced third and fourth peaks. There may be further peaks; however, the small box size does not allow us to get a reliable estimate of the water structure beyond the fourth peak. The HDL only shows three peaks with much less structure. It is worth noting that not only do the second and third peaks of HDL reside at shorter distances but also the HDL second and third peaks have similar heights, whereas the LDL shows a clear progression with each subsequent peak lower than the proceeding one.

**Figure 5:**
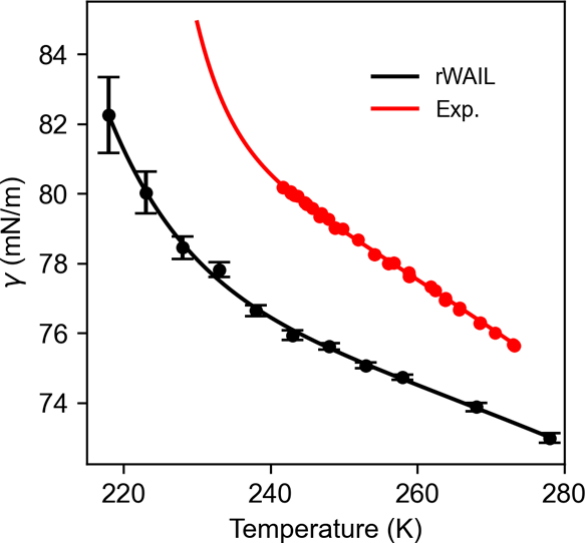
Surface tension for rWAIL (computed with the EG273 variant) and from experiments.^29^ Symbols are data points, and the lines are fits to the IAPWS-E equation.

It has been proposed previously based on the WAIL model that the γ of supercooled water shows an exponential divergence at lower temperatures.^28^ The emergence of the exponential term was later confirmed by experiments.^29^ This divergent behavior has been attributed to the emergence of the LDL. Table 4 and Figure 5 show the fit to the IAPWS-E equation with the exponential term. The experimental fit was performed using the most recent γ of supercooled water obtained with the capillary rise method from −31.4 C^29^ along with the γ of normal water up to 473 K.

**Table 4:** Fitted Parameters for the IAPWS-E Equation for Each Model and Experimental Data

	*T*_c_ (K)	μ	*B* (mN*m^–1^)	*b*	*c* (K^–1^)	*T*_e_ (K)
WAIL	711	11/9	265.926	–0.758	0.0766	230.703
rWAIL^a^	683	11/9	243.057	–0.727	0.0872	237.653
Exp.	647.096	11/9	223.853	–0.587	0.2156	235.861

a Simulated using the EG273 version of rWAIL.

Although the rWAIL model underestimates the experimental γ by roughly 2 mN/m in this temperature range, the trend predicted by rWAIL is similar to the fit to experiments. The *T*_e_ of the rWAIL model, as reported in Table 4, differs only by 2 K from the fit to experiments, which is a big improvement over the WAIL model. The good agreement of *T*_e_ suggests that rWAIL predicts an exponential growth similar to that observed experimentally, which is likely a result of the emergence of LDL. This adds confidence to the LLCP prediction of the rWAIL model. We thus anticipate that real water would have the LLCP close to 203 K and 90 MPa.

Although rWAIL shows a higher *P*_C_^LL^ when compared to WAIL, it is still less than the prediction of most other empirical models, which usually place the LLCP at above 200 MPa. The location of the LLCP at 203 K and 90 MPa with a density of 980 kg/m^3^ is in good agreement with a recent polynomial fit to experimental density data by Mishima,^67^ which suggest the *P*_C_^LL^ to be 105 MPa, a *T*_C_^LL^ of 207 K, and a ρ_C_^LL^ of 993 kg/m^3^. The agreement in the liquid–liquid critical temperature and density is both within 2%.

## 5 Summary and Conclusions

In this study, the rWAIL model was fitted using the same reference forces and the same energy expressions as the WAIL model, with the only change being the use of a SAPT-based dispersion. The new dispersion amounts to roughly a 20% reduction of the C_6_ parameter. Such a seemingly minor difference leads to a model that shows significant improvement in most properties compared to the old WAIL model. Our data suggest that the minor revision in the dispersion mostly affects densities and pressures.

The rWAIL model compares favorably to the ab initio-based MB-Pol model, although the cost of simulation of rWAIL is orders of magnitude smaller. Gromacs input files for rWAIL simulations are provided as the Supporting Information. All variants of the rWAIL model can be simulated with most standard MD programs. At ambient temperature, the rWAIL model provides dielectric constant and surface tension in better agreement with experiments than MB-Pol. Assuming quantum nuclear corrections increase the diffusion constants by 15%, the rWAIL model is also likely to be better in terms of modeling the self-diffusion of water. On the other hand, near the liquid–vapor critical point, the explicit modeling of many-body effects leads to better agreements of MB-Pol in terms of the critical temperature and density. The MB-Pol model also gives better agreement with experiments in regard to isothermal compressibility. The relative accuracy of the two models in reproducing other properties is more difficult to assess due to the lack of proper modeling of quantum nuclear effects or uncertainties in simulation methods. Overall, we believe that the rWAIL model is competitive to the more sophisticated MB-Pol model for simulations of liquid and ice at or below the ambient temperature, whereas the latter is more competitive when computational cost is not a concern or when the vapor phase is involved.

The rWAIL model clearly shows an LLPT with an LLCP at 203 K and 90 MPa, which is in good agreement with recent experimental extrapolations of Mishima.^67^ The rWAIL *T*_C_^LL^ and ρ_C_^LL^ both agree with the Mishima fit within 2%. Also, the rWAIL model continues to show the emergence of an exponential divergence of the γ of supercooled water. The fit to the IAPWS-E equation showed a *T*_e_ of 238 K for the rWAIL model, which agrees with the fit from experimental γ within 2 K.

The coupled cluster-based rWAIL model has no adjustable parameters. The model seamlessly joins two independent pieces of experimental evidence related to the LLPT in supercooled real water. Namely, the model agrees with both the fit to the recent γ measurements and the polynomial fit to the density. Thereby, the fits to rWAIL paint a unified picture in that both experimental fits are consistent with a single first-principles-based model, which strongly supports the existence of a liquid–liquid phase transition in real water, with an LLCP near 203 K and 90 MPa.

## Supporting Information Available

Graph showing the Wegner expansion of the rWAIL model for liquid–gas critical point determination (PDF)
Input files for all three variants of the rWAIL models discussed in the text (ZIP)
