# Supplementary material for: Water Potential from Adaptive Force Matching for Ice and Liquid with Revised Dispersion Predicts Supercooled Liquid Anomalies in Good Agreement with Two Independent Experimental Fits
Source: J Phys Chem B. 2024 Mar 27;128(14):3398–407. doi: 10.1021/acs.jpcb.3c06495 (PMC11017247; doi:10.1021/acs.jpcb.3c06495)
Supplement: Supplementary file 1 — jp3c06495_si_001.pdf [file jp3c06495_si_001.pdf]

Supporting Information (SI)

Water Potential from Adaptive Force Matching for Ice and Liquid with Revised Dispersion Predicts Supercooled Liquid Anomalies in Good Agreement with Two Independent Experimental Fits

Raymond Weldon[a], Feng Wang[a]*

[a] *Department of Chemistry and Biochemistry, University of Arkansas, Fayetteville, AR 72701, USA*

*Email: fengwang@uark.edu

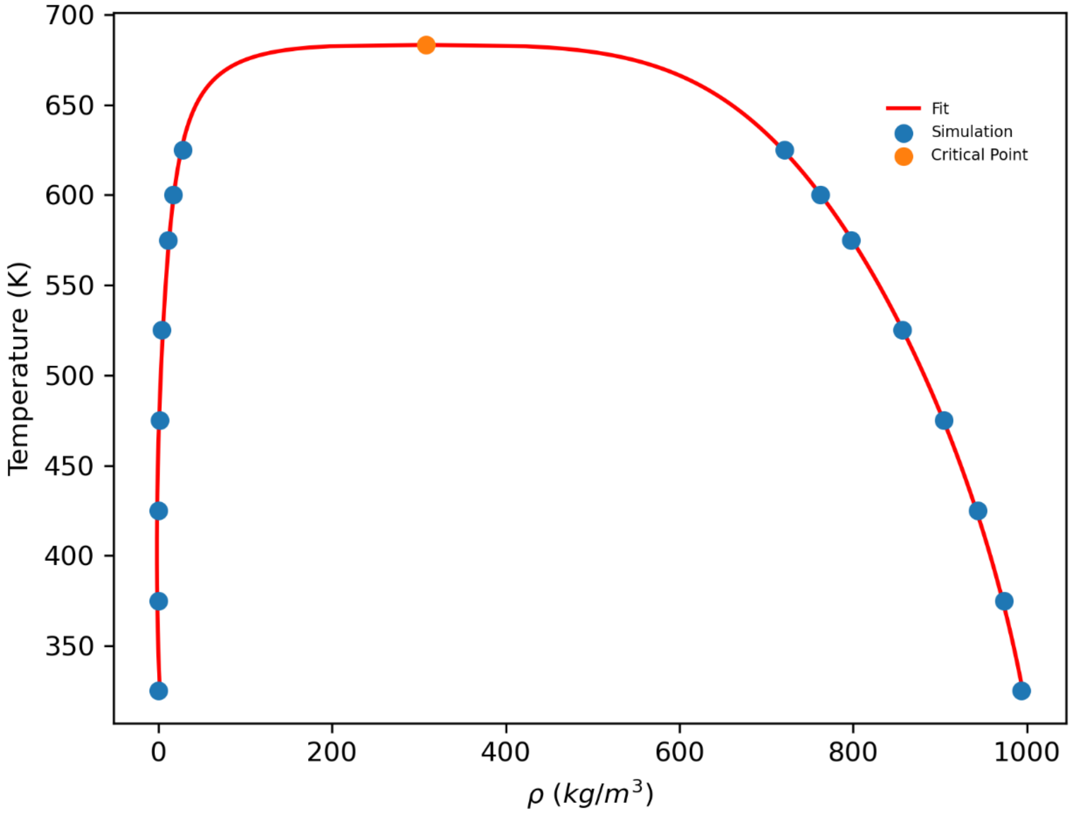

Figure S1: Fit of the liquid and gas densities of rWAIL water to the Wegner expansion. (Computed with the EG273 variant.)

The Gromacs input files for flexible rWAIL and the EG273 and EG298 are provided as supporting information to this manuscript. They are also available at https://wanglab.uark.edu/Models
